# Explaining Waldorf students’ high motivation but moderate achievement in science: is inquiry-based science education the key?

**DOI:** 10.1186/s40536-021-00107-3

**Published:** 2021-06-23

**Authors:** Silvia Salchegger, Christina Wallner-Paschon, Christian Bertsch

**Affiliations:** 1 IQS-Federal Institute for Quality Assurance of the Austrian School System, Alpenstraße 121, 5020 Salzburg, Austria; 2 University College of Teacher Education Vienna, Grenzackerstraße 18, 1100 Wien, Austria

**Keywords:** Inquiry-based science education, Waldorf education, PISA science achievement, Enjoyment of science, Interest in science

## Abstract

**Background:**

Evidence so far shows that Waldorf students are characterized by average science achievement but at the same time high socioeconomic status and high science motivation. Moreover, Waldorf education is characterized by high emphasis on inquiry-based science education (IBSE). The present study investigates if the specific attitude-achievement constellation of Waldorf students in science may be explained by the high level of IBSE.

**Methods:**

Propensity score matching was applied to control for Waldorf students’ more advantageous social background using the Austrian PISA 2015 sample (N = 7007 15-year old students). After propensity score matching, 1107 matched controls were included alongside all 149 Waldorf students.

**Results:**

The results show that Waldorf students report higher enjoyment in learning science and more interest in broad science topics than matched controls. On the other hand, they demonstrate lower science achievement. Mediation analyses show that, although Waldorf students’ more positive attitudes towards science can be almost entirely attributed to their higher exposure to IBSE, their underperformance in science cannot.

**Conclusions:**

These results indicate that attending a school type with a high level of IBSE (Waldorf) may have positive effects on attitudinal outcomes (enjoyment and interest in science) whereas it does not seem to have notable effects on science achievement. This indicates that IBSE could be applied in educational contexts aiming to increase students’ scientific attitudes.

## Background

In today’s world, scientific literacy is seen as essential for individuals to fully participate in society (OECD, 2016b). Many societal decisions (e.g., how do we address the challenge of global warming?) but also personal issues (e.g., will I apply for a Covid-19-vaccination?) are based on a basic understanding of scientific concepts. Additionally, a population with high science skills promotes technological innovation and economic growth of a country (Hanushek & Woessmann, 2012). However, there is growing concern that technological growth will be hindered by a lack of young adults who decide for a career in science and technology (OECD, 2008; Osborne, 2003). This is where students’ attitudes come into play: There is broad evidence that only students who are interested in science and who enjoy and value science will actually go for a career in science and technology (OECD, 2008; Renninger et al., 2015; Schiepe-Tiska et al., 2016; Wang et al., 2013).

Therefore, innovative curricula and ways of science teaching that address the issue of low student motivation needs to be identified and evaluated (Osborne & Dillon, 2008). One specific way of teaching science that seems promising in raising students’ interest in and enjoyment of science is inquiry-based science education (IBSE).

A school type that has a long tradition in teaching science according to methods which are now called “inquiry-based” are Waldorf schools. This leads to the central question of the present paper: Can the Waldorf style of science teaching with its high emphasis on IBSE promote students’ science achievement and science motivation?

### Inquiry-based science education (IBSE): definitions

IBSE aims to provide students with knowledge via investigation, rather than receiving knowledge directly from teachers (Lazonder & Harmsen, 2016). One key element of various definitions of IBSE is that students engage actively (hands-on and minds-on) in the learning process with emphasis on observations and experiments as sources of evidence for defensible conclusions. The hands-on–minds-on notion is also reflected in the definition of PISA (OECD, 2016a, p. 70): *“Inquiry based science education is about engaging students in experimentation and hands-on activities, and also about challenging students and encouraging them to develop a conceptual understanding of scientific ideas.”*

The following definition of the National Science Education Standards (NSES) breaks IBSE down to specific activities and has often been employed in literature as well as directly informed the development of the PISA questionnaires (cf. Jerrim et al., 2020):

*Inquiry is as multifaceted activity that involves making observations; posing questions; examining books, and other sources of information to see what is already known; planning investigations; reviewing what is already known in light of experimental evidence; using tools to gather, analyze, and interpret data; proposing answers, explanations, and predictions; and communicating the results (NRC, *1996*, p. 23).*

It is difficult to exactly trace the first appearance of IBSE. John Dewey, Jean Piaget, and Lev Vygotsky anticipated many aspects of IBSE in their work about the nature of learning and teaching, like the need to motivate students with hands-on activities and provide opportunities for engaging in active thinking and drawing conclusions from data they have gathered on their own. In 1910 John Dewey criticized that “*science has been taught too much as an accumulation of ready-made materials with which students are to be made familiar and not enough as a method of thinking*” (Dewey, 1910, p.122). Dewey placed inquiry at the center of his educational philosophy. For him, learning was best approached by engaging student communities in an inquiry process (Dewey, 1933).

While IBSE is not especially new in science education it has been increasingly debated in reform documents over the last 20 years. In 1996 the US National Science Education Standards (NSES) declared that inquiry is central to science learning (National Research Council, 1996). In 2007 the European Commission demanded a reversal of school science-teaching pedagogy from mainly deductive to inquiry-based methods. In 2018 one can find the term inquiry in almost any curriculum in industrialized countries—from primary to higher education, and it is also endorsed by many funding agencies for grants, for instance the EU Horizon 2020.

### Effectiveness of IBSE

Even though IBSE has become a highly valued and recommended instructional approach (Artigue et al., 2012; European Commission, 2007; Flick & Lederman, 2006; National Research Council, 2000, 2012), its efficacy has been continually challenged.

Data from PISA 2015 show, for the 35 OECD countries, that 15-year-old students who report higher exposure to IBSE do not score higher in science than their peers. Worse even, in 25 of these countries, greater exposure to IBSE was negatively associated with science performance (OECD, 2016a, p. 303). Still, the mean correlation across OECD countries was low (*r* = − 0.08; *p* < 0.01; own computation based on the international PISA 2015 database). Austria was among the ten OECD countries where the relationship between IBSE and science achievement was not statistically significant, however it became significantly negative after controlling for social background indicators.

Although the effect size is small, the negative association between IBSE and student achievement found in PISA 2015 has caused a lot of discussion in the science education research community (Osborne et al., 2017; Sjøberg, 2017) as well as deeper analysis of the PISA data. A recent publication on PISA 2015 (Mostafa et al., 2018) showed that the negative association between IBSE and science performance disappears when lessons are adequately designed to compensate for students’ lack of experience with scientific methods and are delivered in a disciplined environment.

Similarly, a secondary analysis of PISA 2015 data showed that students who receive a blend of inquiry-based along with an adequately supported teacher-directed instruction have the best achievement outcomes (Denoël et al., 2017, p. 40). It must be noted however that in this study teacher-directed instruction refers to science instruction in general and not specifically to the IBSE parts. Therefore, it is unclear whether the IBSE episodes of instruction include the same amount of teacher-directedness as the other parts of science instruction (especially when IBSE makes up only a small part of science instruction). This is a central shortcoming of the Denoël et al. study as Lazonder and Harmesen (2016) as well as Furtak et al. (2012) showed that teacher guidance is of central importance in IBSE. Specifically, teacher-led IBSE activities had a much higher positive impact on learning outcomes than student-led IBSE activities.

Furtak et al. (2012) also showed that teaching approaches with a focus on the epistemic domain of inquiry (nature of science, drawing conclusions based on evidence) had a particularly large effect on science learning. Similarly, Minner et al. (2010) concluded, in their synthesis of 138 studies, that the evidence of positive effects of IBSE on science achievement was only conclusive when the inquiry-based instructional practices emphasized student active thinking and drawing conclusions from data.

Regarding the relationship between IBSE and motivational outcomes it was shown a universal positive relationship between the amount of IBSE and students’ enjoyment of science ranging between r = 0.20 and 0.30 in most countries (Mostafa et al., 2018). Similar positive but weaker associations are observed between interest in broad science topics and exposure to IBSE (Mostafa et al. 2018). Correlations vary between 0.10 and 0.20 in magnitude for most countries.

In sum, a considerable problem in the investigation of the effectiveness of IBSE is that the type of instruction characterized as *inquiry-based* varies greatly (e.g., Furtak et al., 2012). In particular, the amount of teacher guidance represents a critical point of high variation. The term IBSE can be used to refer to instructional approaches with minimal guidance, which has been shown to be less effective (Kirschner et al., 2006), as well as more teacher-centered lessons, where the students conduct activities (supported by the teacher) and draw conclusions from data to answer questions posed by the teacher (Hmelo-Silver et al., 2007). Additionally, the level of teacher guidance cannot be determined for most IBSE items of the PISA scale.

Moreover, existing results on PISA are based on correlation-based analyses. The OECD (2016a) advised interpreting this correlational evidence with caution as the direction of the effect is not clear—for instance, teachers may be using hands-on activities to make science more attractive to disengaged students. Nevertheless, the OECD points out that the constant negative association across so many countries—and even after controlling for social background (cf. Mostafa et al., 2018)—suggests taking concerns about hands-on activities in science class seriously.

However, many of the (quasi-)experimental investigations into the effectiveness of IBSE performed so far have limitations. These limitations concern the varying operationalization of IBSE (e.g., Furtak et al., 2012; Minner et al., 2010), the short time span covered in many (quasi-) experimental studies (e.g., Cobern et al., 2010; Echevarria, 2003), a lack of differentiation between cognitive and attitudinal student outcomes (as in Furtak et al., 2012), and/or very narrow outcome measures (e.g., understanding the concepts of “floating and sinking” as in Hardy et al., 2006). Therefore we lack information about the effects of long-term curriculum-based IBSE on different forms of learning outcomes, including achievement and motivational measures.

The present study aims to close this research gap by investigating whether students attending a school type with high emphasis on IBSE in the curriculum demonstrate higher science achievement and more positive attitudes towards science than non-Waldorf students with similar background characteristics.

We include attitudinal outcomes (enjoyment of learning science and interest in broad science topics) in our analysis because developing positive science-related attitudes is an important educational goal alongside fostering science achievement (e.g., Osborne, 2003; Schiepe-Tiska et al., 2016).

### Waldorf schools

Waldorf schools represent a school type with high emphasis on IBSE. Globally available, this private school system follows concepts outlined by Rudolf Steiner (1861–1925). The school system is rooted in Steiner’s spiritual philosophy: *Anthroposophy*. Accordingly, Waldorf education places high emphasis on students’ creative, social, and emotional development. Moreover, it is characterized by various specifics of instruction (cf. Dahlin, 2017; Steiner, 2003). The following characteristics comprise the most striking differences between Waldorf education (which is the same globally) and education in other^1^ schools:

Unlike other students, Waldorf students do not receive graded marks until the end of Grade 12. Instead, Waldorf students are given individual reports at the end of each school year (Hellmich, 1999). In these individual reports, a student is only compared to his or her past achievement, rather than a general standard or norm. The marks given in Grade 12 are those formally required by the public merit system (Dahlin, 2017, p. 92). Students attending Austrian public schools usually receive grades from first grade or by Grade 4 at the latest (BMBWF, 2018). Moreover, public school students have to repeat a class if they fail to obtain the pass mark in a subject, which is not the case for Waldorf students. Waldorf students also receive the key subjects in blocks, focusing on a certain interdisciplinary issue for longer period within a given timeframe (usually 2 h a day over the period of 1 month; Rauthe, 1992). Science subjects are also part of such main lesson blocks. In comparison, non-Waldorf students in Austria experience lesson blocks of 50 min daily over the entire school year. Moreover, these are taught by specialised teachers from Grade 5 on, whereas Waldorf students retain a general class teacher up to Grade 8 (Rittelmeyer, 2010). Moreover, Waldorf schools tend away from text books.

Waldorf science instruction is characterized by the transition from a real-life perspective (e.g., experiments) to an objectifying one (Masters, 1992). The methodical principle is based on a three-phased structure devised by Steiner (1986, p. 45). The first phase is the observation of a scientific phenomenon. This is often in the form of experiments designed to stimulate students’ independent thinking (Østergaard et al., 2008) and enable real-life comparison. Initially the students observe the phenomena without any background information (e.g., theory, definitions). An example for this phase would be observing the refraction of light through a prism while consciously refraining from theorizing about it (Dahlin, 2009, p. 551). The second phase is characterised by reconstructing the observed phenomenon to enable active perception or active thinking. Here the students are asked to describe, specify, and reflect on their experiments and observations (Krämer et al., 1987; Trostli, 1995). This reflective process leads to concept formation (third phase). In this final stage, the students should systematically arrange the phenomena (e.g., correlations, causes, and effects of the phenomena, theory, definitions) to achieve their cognitive learning objective (Sommer, 2014).

In sum, Waldorf education is characterized by a high emphasis on the observation and inquiry process. The correctness of an answer is evaluated mainly with respect to the available observation and evidence. The teacher does not perform as the sole bearer of expert knowledge, but provides opportunities to inquire, observe, and negotiate ideas. Indeed a very high prevalence of student investigations in science instruction has been reported by Waldorf students in PISA 2006 (Wallner-Paschon, 2009, p. 397).

### Comparison of science-related learning outcomes between Waldorf students and other students

Concerning motivational-affective student outcomes, it was found that Waldorf students report decisively more enjoyment of learning science, more interest in broad science topics, and a higher science self-concept than other students (e.g., Wallner-Paschon, 2009, 2018). Concerning cognitive outcomes, it was shown that Waldorf students slightly outperform other Austrian students in reading and science but they perform slightly below the population mean in math (Wallner-Paschon, 2018). Considering that Waldorf students’ socioeconomic status (SES) is decisively higher than other students’ SES (Wallner-Paschon, 2018), these results indicate underachievement in Waldorf students as their actual achievement is lower than expected by their SES. Studies that control for the higher SES of Waldorf students when comparing achievement outcomes between Waldorf and non-Waldorf students are missing so far. Also, Dahlin (2017, p. 127) criticized that “there are no attempts to measure the influence of students’ SES and other aspects of their family background, to ascertain to what extent the findings are specifically related to the Waldorf methods of education”.

### The present study

The present study investigates—specifically for the subject of science—whether the underachievement but high motivation of Waldorf students may be explained by the high emphasis on IBSE in Waldorf education. As far as we know, it is the first study that examines whether students in a school type with high curricular emphasis on IBSE (Waldorf schools) differ in their science achievement and attitudes towards science from non-Waldorf students. This will add a new facet to IBSE research by showing whether IBSE affects science-related learning outcomes when it is a general curriculum-based educational approach that is (a) not only applied for a limited time-span and restricted to specific topics (like in [quasi-]experimental studies) and (b) not only applied in reaction to low student engagement and/or achievement.

Moreover, it gives us the unique possibility to find out more about achievement and motivation of Waldorf students as Austria is the only country which expanded its PISA sample by a census of Waldorf students. Therefore all 15-year old Waldorf students in Austria were tested by the same PISA instruments as the sampled non-Waldorf students.

Provided Waldorf students and non-Waldorf students are matched by relevant covariates (gender, immigration background, parental education, parental occupational status, and cultural possessions at home), we propose the following hypotheses for the present study:

1. Waldorf students report about more IBSE than other students.
2. IBSE is a mediator of the relationship between Waldorf school attendance and science achievement.
3. IBSE is a mediator of the relationship between Waldorf school attendance and enjoyment of science.
4. IBSE is a mediator of the relationship between Waldorf school attendance and interest in broad science topics.

## Methods

### Sample

The present sample is based on the Austrian PISA 2015 sample. PISA assesses country-representative samples of 15-year old students.^2^ In Austria all 15-year old students from all ten Austrian Waldorf schools were added to the PISA sample, thus achieving a census of 15-year old Waldorf students in Austria. This resulted in a PISA sample of 6858 non-Waldorf students from 259 schools and 149 Waldorf students from ten schools.

The PISA 2018 database (including all Austrian data) is available under https://www.oecd.org/pisa/data/2018database/. IDs of Waldorf schools are available upon request from fdb@iqs.gv.at.

Waldorf students differ from other Austrian students in various background characteristics (Table 1). The differences are specifically large (Cohen’s *d* > 0.65) in indicators of social background (i.e., parental education and occupational status as well as cultural possessions at home) in the direction that Waldorf students on average come from a more advantageous social background.

**Table 1:** Univariate findings before matching for covariates (PISA 2015; *N* = 7007)

Construct	WS (*N* = 149)		Non WS (*N* = 6858)			% Missing values before imputation	
	*M*	*SD*	*M*	*SD*	*d* (WS-RS)	WS	non WS
Covariates for propensity score matching							
Gender (1 = female/0 = male)	0.55 (0.04)	0.50	0.50 (0.01)	0.50	0.11	0	0
Immigration background (1 = yes/0 = no)	0.17 (0.03)	0.38	0.20 (0.01)	0.40	− 0.08	1.31	1.33
Index of parental education in years of schooling	15.73 (0.20)	2.39	14.05 (0.05)	2.47	0.68	2.60	2.50
Index of parental occupational status	67.51 (1.42)	17.13	50.77 (0.47)	21.19	0.79	1.94	6.35
Index of cultural possessions at home	1.06 (0.09)	1.06	0.09 (0.02)	1.03	0.94	0.65	1.44
Analysis variables for mediation modeling							
Inquiry-based science instruction	0.68 (0.09)	0.95	− 0.30 (0.03)	1.09	0.89	14.84	21.13
Enjoyment of science	0.26 (0.09)	1.10	− 0.34 (0.02)	1.25	0.48	6.83	7.34
Interest in broad science topics	0.53 (0.09)	0.72	0.05 (0.06)	0.99	0.48	9.71	10.11
Science achievement	503.30 (6.57)	75.98	495.02 (2.44)	97.38	0.09	0	0

Results are based on imputed manifest scores weighted by PISA total student weight. Standard errors are in parentheses. Cohen’s *d* is computed relative with the *SD* of the weighted total Austrian PISA 2015 sample before matching. Scores for science achievement are based on plausible values. Scores for all other analysis variables are based on Warm’s Mean Weighted Likelihood Estimates (WLE). WLEs and the index of cultural possessions at home were z-standardized based on all OECD countries (OECD *M* = 0, *SD* = 1). Plausible values were standardized across the OECD countries in 2006 (*M* = 500; *SD* = 100). The index of parental occupational status has a minimum of 10 and a maximum of 89

*WS* Waldorf students

### Measures

#### Enjoyment of learning science

Enjoyment of science reflects students’ attachment to learning science and experiencing it as a meaningful activity (Laukenmann et al., 2003). The PISA *enjoyment of learning science* scale was derived from students’ level of agreement with the following items: “I generally have fun when I am learning science topics”, “I like reading about science”, “I am happy working on science topics”, “I enjoy acquiring new knowledge in science”, “I am interested in learning about science”. Students responded on a four-point Likert scale with the categories “strongly agree”, “agree”, “disagree”, and “strongly disagree”. Scaling was performed on item response theory. This process resulted in weighted likelihood estimates (WLEs). For the scale scores provided in the international PISA 2015 database, WLEs were standardized based on all OECD countries. For the present mediation analyses scale scores were standardized based on the weighted total Austrian sample. The scale is polled in a way that higher scores indicate more enjoyment. The reliability of this scale was excellent in Austria (ρ = 0.95; OECD, 2017). The missing rate is 7% for both Waldorf students and non-Waldorf students (see Table 1). This scale was first assessed in PISA 2006.

#### Interest in broad science topics

Interests reflect enduring patterns of motivation for pursuing context-specific activities, outcomes, and environments (Hoff et al., 2018). The PISA 2015 scale *interest in broad science topics* was derived from student’s indication of interest with the following topics: “Biosphere (e.g., ecosystem services, sustainability)”, “Motion and forces (e.g., velocity, friction, magnetic and gravitational forces)”, “Energy and its transformation (e.g., conservation, chemical reactions)”, “The Universe and its history”, “How science can help us prevent disease”. For each topic, students declared their interest on a five-point Likert scale with the categories “not interested”, “hardly interested”, “interested”, “highly interested”, and “I don’t know what this is”. The last category was recoded as missing. Scaling and standardization were performed the same way as described for the enjoyment of learning science scale. Higher scores indicate more interest. The reliability of this scale was ρ = 0.77 in Austria (OECD, 2017). The missing rate is 10% for both Waldorf students and non-Waldorf students (see Table 1).

In PISA 2006 a scale named *interest in science learning* was assessed. Although this scale also covers interest in science, it is not comparable to the 2015 scale as it was based on different items (cf. OECD, 2009a, p. 319). It may however show whether the difference of science interest between Waldorf and non-Waldorf students keeps stable over time (see Table 2).

**Table 2:** Univariate findings for PISA 2006 (*N* = 5080)

Construct	WS (*N* = 153)		Non WS (*N* = 4927)		
	*M*	*SD*	*M*	*SD*	*d* (WS-MS)
Student background					
Gender (1 = female/0 = male)	0.58	0.49	0.49	0.50	0.18
Immigration background (1 = yes/0 = no)	0.11	0.31	0.13	0.34	− 0.08
Index of parental education in years of schooling	15.41	2.03	13.75	2.33	0.71
Index of parental occupational status	62.11	15.44	48.31	16.58	0.83
Index of cultural possessions at home	0.84	0.73	0.07	0.96	0.80
Instruction and outcome variables					
Enjoyment of science	0.41	0.94	-0.21	1.10	0.57
General interest in learning science PISA 2006	0.52	0.74	0.05	0.89	0.53
Science achievement	523.90	81.50	510.84	97.86	0.13
Single IBSE variables assessed in both PISA 2006 and 2015 (percentage of students who report that this happens in most or in all science lessons)					
Students are given opportunities to explain their ideas (2006)	0.86	0.35	0.54	0.50	0.63
Students are given opportunities to explain their ideas (2015)	0.85	0.36	0.62	0.48	0.47
Students spend time in the laboratory doing practical experiments (2006)	0.65	0.48	0.16	0.37	1.32
Students spend time in the laboratory doing practical experiments (2015)	0.66	0.48	0.18	0.38	1.25
Students are asked to draw conclusions from an experiment they have conducted (2006)	0.83	0.38	0.38	0.49	0.92
Students are asked to draw conclusions from an experiment they have conducted (2015)	0.81	0.40	0.28	0.45	1.17
Students are allowed to design their own experiments (2006)	0.27	0.45	0.12	0.33	0.45
Students are allowed to design their own experiments (2015)	0.24	0.43	0.11	0.31	0.44

Results are based on imputed manifest scores weighted by PISA total student weight. Cohen's *d* is computed relative with the *SD* of the weighted total Austrian PISA 2015 sample before matching. Scores for science achievement are based on plausible values, scores for all other instruction and outcome scales are based on Warm's Mean Weighted Likelihood Estimates (WLE)

*WS* Waldorf students

#### IBSE

Students were asked how frequently (“in all lessons”, “in most lessons”, “in some lessons”, and “never or hardly ever”) the following happens in their science lessons: “Students are given opportunities to explain their ideas*”, “Students spend time in the laboratory doing practical experiments*”, “Students are required to argue about science questions”, “Students are asked to draw conclusions from an experiment they have conducted*”, “The teacher explains how a science idea can be applied to a number of different phenomena (e.g., the movement of objects, substances with similar properties)”, “Students are allowed to design their own experiments*”, “There is a class debate about investigations”, “The teacher clearly explains the relevance of science concepts to our lives”. Items were reverse coded for scaling so that a higher scale score indicates more IBSE. Scaling and standardization were performed the same way as described for the enjoyment of learning science scale. The reliability of this scale was ρ = 0.87 in Austria (OECD, 2017).

It must be noted that the amount of missing data in the original data is rather high for the IBSE scale: 15% in Waldorf students and 21% in other students. However the missing rate in non-Waldorf students who were matched to Waldorf students (and therefore included into the present mediator analyses) was decisively lower (12%). The four IBSE items indicated by an asterisk above have already been assessed in PISA 2006. Table 2 (bottom part) gives a 9-year-trend for these items.

#### Science proficiency scale

PISA defines science as “the ability to engage with science-related issues, and with the ideas of science, as a reflective citizen” (OECD, 2017, p. 43). Scientific literacy encompasses the competencies to explain phenomena scientifically, to evaluate and design scientific inquiry, and to interpret data and evidence scientifically. All of these competencies are covered by the PISA science scale. Scaling was performed using item response theory. This process resulted in ten plausible values (PVs), which are provided in the international PISA 2015 database. There are no missing values in the PVs. The reliability of the PISA science scale was excellent in Austria (ρ = 0.83; OECD, 2017). Released science items may be found under https://www.oecd.org/pisa/38709385.pdf.

### Social and cultural background

The following indicators of social and cultural background were used to match non-Waldorf students to Waldorf students:

#### Immigration background

Students whose parents were both born abroad were coded as students with an immigration background (cf. OECD, 2016b).

#### Index of parental education in years of schooling

PISA classified students’ responses on questions regarding parental education using the *International Standard Classification of Education* (ISCED) 1997 (OECD, 1999). ISCED Codes were then mapped to years of education (see OECD, 2017, p. 435, for the mapping scheme).

The *index of parental occupational status* was derived from students’ responses on their parents’ occupation via coding occupations according to the International Standard Classification of Occupations based on prestige and income of occupations. The resulting index ranges from 10 (e.g., kitchen helpers) to 89 (medical doctors; cf. Ganzeboom, 2010).

The *index of cultural possessions at home* was derived from students’ reports on the amount of the following cultural possessions at home: “classic literature”, “books of poetry”, “works of art (e.g., paintings)”, “books on art, music, or design”, “musical instruments (e.g., guitar, piano)”. Resulting WLEs were standardized based on all OECD countries.

### Data analysis

#### Sampling and replicate weights

Descriptive statistics were performed using the IEA IDB Analyzer (IEA, 2019). This program takes into account sample weights (yielding population-representative results), replicate weights to account for the two-stage sample design (yielding adjusted standard errors) as well as plausible values (yielding unbiased achievement results). In this way, the special computation requirements for PISA data (cf. OECD, 2009b) are met.

#### Generalizability of Waldorf students’ characteristics across different cohorts

Although the Austrian PISA 2015 sample includes the whole census of 15-year old Austrian Waldorf students, the total number of Waldorf students is rather small (N = 149 students). In order to test for the generalizability of Waldorf student characteristics across different cohorts, we compared the same or similar constructs between PISA 2006 and PISA 2015. Results indicate a high stability of differences between Waldorf and non-Waldorf students in the outcome variables enjoyment of science (2006 *d* = 0.57; 2015 *d* = 0.48) interest in science (2006 *d* = 0.53; 2015 *d* = 0.48) and science achievement (2006 *d* = 0.13; 2015: *d* = 0.09), each to the advantage of Waldorf students (Table 2 in comparison to Table 1). Results on the single IBSE items show a high stability in the 9-year trend for Waldorf students and even a higher stability than for non-Waldorf students (Table 2). This indicates that (a) inquiry-based instruction kept probably more stable for Waldorf students than for non-Waldorf students in the time span between 2006 and 2015 and (b) it is highly feasible that the rather small sample size of Waldorf students did *not* result in random fluctuations.

#### Invariance testing

We performed confirmatory factor analysis based on weighted least squares estimation which is the currently recommended estimator for ordinal data (Bowen & Masa, 2015) to test that the constructs IBSE, enjoyment of science and interest in broad science topics were measured the same way across Waldorf and matched non-Waldorf students. Results show that the models testing for metric and for scalar invariance fit the data adequately and about to the same extent (CFI = 0.975; RMSEA = 0.067 [90% CI 0.057, 0.077], for metric invariance). This indicates that the same constructs were measured in Waldorf and non-Waldorf students.

#### Multiple imputation

Multiple imputation is currently considered the preferable approach for dealing with missing data to avoid biased estimates (Enders, 2010; Schafer & Graham, 2002). The multiple imputation procedure provided by IBM SPSS Statistics 24 was used to replace missing data. This software uses the sequential regression approach as the algorithm for multiple imputation (Enders, 2010). All covariates in the propensity score model, the analysis variables for mediation modeling (see Table 2), and some additional correlated variables (i.e., reading achievement, math achievement) were included in the imputation model. In total, we imputed 10 data sets, which were further analyzed separately. All parameter estimates and standard errors were combined by Rubin’s (1987) rules.

#### Propensity score matching

Propensity score matching was used as a pre-processing strategy to control for systematic differences between Waldorf students and non-Waldorf students on various background characteristics. This approach has frequently been used in education research when investigating the effectiveness of different tracks (e.g., Becker et al., 2012; Guill et al., 2017) and is a recommended procedure for observational data when no randomly selected group samples are available (Stuart, 2010). If subject groups can reasonably be regarded as statistically equivalent on relevant covariates, average differences on the outcomes could plausibly be attributed to the treatment (Rutkowski, 2016). For the present study this means that, after statistical equivalence in background characteristics is established, average differences on science achievement and science motivation can plausibly be attributed to Waldorf science instruction. It must be noted however, that complete equivalence is difficult to achieve as a number of relevant covariates (e.g., prior achievement, parental educational goals) are not assessed in PISA. Moreover, there is need to focus on a limited number of relevant covariates because the higher the number of covariates is the more difficult it becomes to find matching partners with high similarity on all aspects.

We included the following covariates into the model: *gender* because it is related to science achievement and motivational outcomes in Austria, *immigration background* because it is strongly related to achievement (Suchan & Breit, 2016), *parental education, parental occupational status,* and *cultural possessions at home* because these are highly related to both achievement and Waldorf school attendance.

We performed propensity score matching in *IBM SPSS Statistics Version 24* using Essentials for R^3^ in combination with the Software *PS Matching in SPSS Version 3.03.*^4^ All covariates except for gender were standardized based on the weighted total Austrian sample before matching. We matched Waldorf students and non-Waldorf students on the following characteristics: gender, immigration background, parental education, parental occupational status, and cultural possessions at home (see covariates in Table 1). Propensity score matching was performed for each of the ten imputed datasets separately. We performed nearest neighbor matching^5^ without replacement and a ratio of 1:1 (i.e., each Waldorf student is matched to exactly one student from the comparison group). Matched pairs did not have to be identical on their propensity score but we allowed for small differences (caliper width of 0.1); we choose random matching in order to increase the diversity of matched students across the ten imputed files. The propensity scores were transformed to logits to normalize the skewed distributions in both groups. The effect size of the difference in means of the propensity scores between Waldorf students and matched controls varies around *d* = 0.05 across the 10 datasets (*Mdn* = 0.055). Also, SDs between Waldorf and other students are very similar in size. This indicates that appropriate matching partners could be found in most cases.

Depending on the data set, no matching partner could be found for 4 to 7 Waldorf students. As mediation analyses based on *type is imputation* can only be performed using Mplus when each dataset includes the same number of cases, we replaced missing matching partners from one dataset with the student’s matching partner from another data set. In the end, only three of 149 lacked a matching partner across all ten imputed datasets. Altogether, 1107 different students from the non-Waldorf sample were matched to the same 146 Waldorf students across all ten datasets. In 353 cases, the same non-Waldorf student was matched to the same Waldorf student in at least two imputed datasets. In sum, Waldorf students are compared to a large sample of non-Waldorf students, increasing the reliability of results. After matching, matched control students received the same sample weight as the respective Waldorf student because matched students should mirror Waldorf student population and not Austrian student population.

#### Single-indicator structural mediation modeling

Mediation modeling is a regression-based approach used to explain the relationship between an independent variable (Waldorf school attendance) and a dependent variable (science achievement or science attitudes) via a third variable, known as the mediator (IBSE). It is used to test whether the observed relationship between the two variables may be explained by a third variable (i.e., the mediator variable). Anderson and Gerbing (1988) recommended a two-step-approach in structural modeling with separate assessment of the measurement model and the structural model. However, if an independent estimate for the error variance is available from prior research, single-indicator structural models can be specified without separate assessment of the measurement model. As reliabilities are known for all of the present scales, we stick to this approach and perform single-indicator structural models in order to test the mediation hypotheses. This is done by fixing the residual variance to the error variance while fixing the factor loading to 1 (as proposed by Wang & Wang, 2012). We corrected for measurement error in all endogenous variables. There was no measurement error in the exogenous variable (Waldorf school attendance).

An advantage of single-indicator structural models is that they require smaller sample size than multiple-indicator latent-variable models. According to Fritz and MacKinnon (2007) a minimum sample size of n = 66 is required for mediation models based on our methods (Sobel test under ML condition) for 0.8 power (which is usually considered adequate in psychology, Cohen, 1990). Our sample size of 295 students per data set (and 1256 in total) is therefore considered more than sufficient for results with adequate power. However, the present sample size could be too small when performing multiple-indicator latent-variable modeling. In this case a sample size that is at least five times as high as the free parameters (in our case 104 × 5 = 520 students per data set; Bentler & Chou, 1987) is recommended.

Structural equation modeling (SEM) was performed in M*plus* Version 7 (Muthén & Muthén, 1998–2012) using ML estimation with robust standard errors. Statistical significance was assessed using a predetermined significance level of α = 0.05, whereas practical significance was assessed by interpreting standardized path coefficients β as effect size measures. According to Cohen (1988), values of |β| ≥ 0.10 may be cautiously interpreted as small effects, |β| ≥ 0.30 as moderate effects, and |β| ≥ 0.50 as large effects. Standardization for all mediation models is based on the total Austrian population (including both Waldorf and non-Waldorf students) of 15-year old students (= weighted PISA sample).

Three single-indicator SEMs were estimated to test Hypotheses 2–4. These models are all mediation models that only differ by the outcome variables. The outcome variable was science achievement in Model 1, enjoyment of learning science in Model 2, and interest in broad science topics in Model 3.

**Fig. 1:**
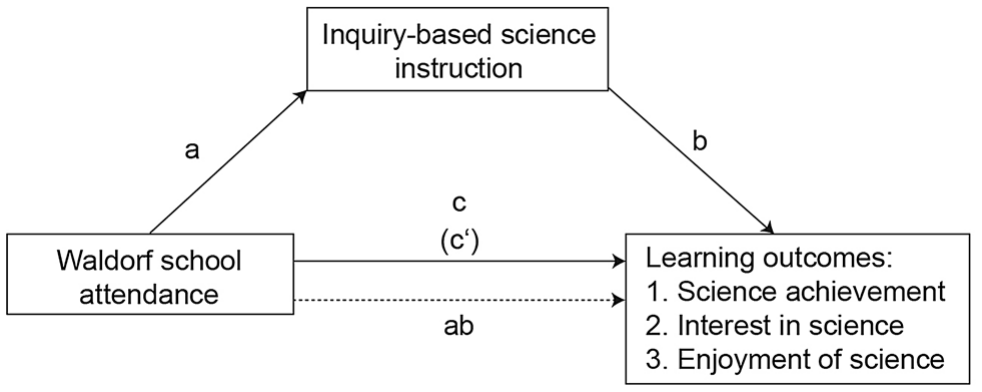
Conceptual model: How inquiry-based science instruction mediates the relationship between Waldorf school attendance and students’ learning outcomes

Figure 1 shows the conceptual model. The total effect *(c)* represents the sum of the indirect and direct effects of Waldorf school attendance on the respective learning outcome (either science achievement, interest in science, or enjoyment of science), where the indirect effect is the product of paths *a* (the effect of Waldorf school attendance on IBSE) and *b* (IBSE on the respective learning outcome), and the direct effect *(cʹ)* is the effect of Waldorf school attendance on learning outcomes after controlling for IBSE. The total effect *(c)* was estimated by regressing science achievement on the respective learning outcome without controlling for IBSE.

In addition, path *ab* is an indirect effect because it links the independent and dependent variables through the mediator. The indirect effect equals the reduction of the effect of the initial variable on the outcome: *ab* = *c* − *cʹ*. The indirect effect is the measure of the amount of mediation in contemporary mediation analyses (Hayes, 2013). Following the guidelines of Preacher and Kelley (2011) we consider a mediation effect as *complete* if *cʹ* is not statistically significantly different from zero.

To account for unreliability in the factor scores, single indicator latent factors were specified for each of the four endogenous variables where the unstandardized factor loading was fixed at 1 and the error variance was set equal to *Var*(X)(1 *−* ρ_x_) (the sample variance of the factor scores multiplied by one minus the estimated reliability of the factor scores; Brown, 2006). Due to standardization the sample variance of the factor scores was 1; therefore the error variance was equal to 1 *−* ρ_x._

## Results

### Propensity score matching

In Table 1 we present sample differences after multiple imputation but before propensity score matching for covariates and outcome variables. Table 3 shows sample differences after 1:1 matching of Waldorf and non-Waldorf students to reveal the effects of matching on group differences. After matching, standardized mean differences of all covariates were below *d* = 0.1 and therefore far below the criterion of *d* = 0.25 for acceptable group differences after matching (Stuart, 2010). Altogether, matching was very successful as group differences in background characteristics were close to zero after matching with the highest remaining difference for home possessions (*d* = 0.08).

**Table 3:** Univariate findings after matching for covariates (PISA 2015; N = 1256)

Construct	WS (*N* = 149)		non WS (*N* = 146)^a^		
	*M*	*SD*	*M*	*SD*	*d*
Covariates for propensity score matching					
Gender (1 = female/0 = male)	0.55 (0.04)	0.50	0.54 (0.04)	0.50	0.02
Immigration background (1 = yes/0 = no)	0.17 (0.03)	0.38	0.18 (0.04)	0.39	− 0.03
Index of parental education in years of schooling	15.73 (0.20)	2.39	15.81 (0.19)	1.80	− 0.03
Index of parental occupational status	67.51 (1.42)	17.13	67.17 (1.69)	18.24	0.02
Index of cultural possessions at home	1.06 (0.09)	1.06	0.98 (0.12)	1.11	0.08
Analysis variables for mediation modeling					
Inquiry-based science instruction	0.68 (0.09)	0.95	− 0.22 (0.12)	1.10	0.82
Enjoyment of science	0.26 (0.09)	1.10	− 0.04 (0.15)	1.22	0.24
Interest in broad science topics	0.53 (0.09)	0.72	0.23 (0.09)	0.99	0.30
Science achievement	503.30 (6.57)	75.98	534.89 (10.12)	92.96	-0.32

Results are based on imputed manifest scores weighted by PISA total student weight. Standard errors are in parentheses. Cohen's *d* is computed relative with the *SD* of the weighted total Austrian PISA 2015 sample before matching. Scores for science achievement are based on Plausible Values; scores for all other analysis variables are based on Warm's Mean Weighted Likelihood Estimates (WLE)

*WS* Waldorf students

^a^146 students per data set and 1107 different students across all 10 data sets

As expected, differences in science achievement increased as a result of matching: While the science achievement gap was *d* = 0.09 in favor for Waldorf students before matching, it was *d* = − 0.32 to the detriment of Waldorf students after matching (see Tables 1 and 3). The advantages of Waldorf students in terms of interest (*d* = 0.30) and enjoyment (*d* = 0.24) however were still remarkable after matching. Moreover, the much higher level of IBSE reported by Waldorf students was still clear after matching (*d* = 0.89 before matching and *d* = 0.82 after matching). This last result confirms Hypothesis 1: Waldorf students report about more IBSE than other students.

### Mediation modeling

Mediation models include all Waldorf students (*N* = 149) as well as the non-Waldorf students matched to Waldorf students by propensity score matching (*n* = 146 in each dataset). As explained under the “Propensity score matching” section, the matched students may vary across datasets.

In a first mediation analysis, we tested if IBSE was a mediator of the relationship between Waldorf school attendance and science achievement (Table 4, Model 1). Results show a nonsignificant indirect effect which is negligible in size (β = 0.07; *p* = 0.383). Therefore, the underperformance of Waldorf students in science cannot be explained by the higher amount of IBSE in Waldorf education. This indicates that there must be other reasons than IBSE which are responsible for the underperformance of Waldorf students (see “Discussion” section). In sum Hypothesis 2 is not supported: IBSE is not a mediator of the negative relationship between Waldorf school attendance and science achievement. Results of further mediation analyses show that IBSE is a significant mediator of the relationship between Waldorf school attendance and students’ enjoyment of learning science (Table 4, Model 2) as well as of the relationship between Waldorf school attendance and students’ interest in broad science topics (Table 4, Model 3). Concerning enjoyment of learning science, the size of the indirect effect is moderate (β = 0.31, *p* = 0.010) and even larger than the total effect: After controlling for IBSE, the direct effect of Waldorf school attendance on science enjoyment is even slightly negative. For interest in broad science topics, the indirect effect is slightly smaller (β = 0.24, *p* = 0.016) than for enjoyment but nevertheless significant. In both models (enjoyment and interest) the direct paths cʹ are nonsignificant and close to zero, which indicates almost complete mediation (Preacher & Kelley, 2011). Referring to the content of our study, the nonsignificant direct paths *c*ʹ in Models 2 and 3 indicate that Waldorf students’ attitudes towards science (enjoyment of science and interest in science) would not be significantly different from those other students without the higher IBSE. This indicates that Waldorf students’ higher science-related enjoyment and interest may almost entirely be attributed to the higher amount of IBSE in Waldorf education. Altogether these results strongly support Hypotheses 3 and 4.

**Table 4:** Mediation effect estimates based on single-indicator latent-variable models

Model	Standardized estimate	*SE*
Model 1: outcome is s*cience achievement*		
Model without mediator		
Waldorf school attendance → outcome *(c)*	− 0.32*	0.12
Model with mediator		
Waldorf school attendance → IBSE *(a)*	0.82*	0.13
IBSE → outcome *(b)*	0.09	0.10
Waldorf school attendance → outcome *(c')*	− 0.40*	0.16
Indirect effect *(ab)*	0.07	0.80
Model 2: outcome is *enjoyment of learning science*		
Model without mediator		
Waldorf school attendance → outcome *(c)*	0.24	0.14
Model with mediator		
Waldorf school attendance → IBSE *(a)*	0.82*	0.13
IBSE → outcome *(b)*	0.37*	0.12
Waldorf school attendance → outcome *(c')*	− 0.07	0.17
Indirect effect *(ab)*	0.31*	0.12
Model 3: outcome is *interest in broad science topics*		
Model without mediator		
Waldorf school attendance → outcome *(c)*	0.30*	0.11
Model with mediator		
Waldorf school attendance → IBSE *(a)*	0.82*	0.13
IBSE → outcome *(b)*	0.30*	0.11
Waldorf school attendance → outcome *(c')*	0.06	0.14
Indirect effect *(ab)*	0.24*	0.10

*SE* standard error

^*^p < 0.05

## Discussion

Waldorf education is characterized by a high amount of curriculum-based IBSE, and indeed a very high amount of IBSE was reported by Waldorf students in the present study. Therefore, Waldorf students are an ideal population for revealing the effects of IBSE. In order to investigate the specifics of Waldorf students in terms of IBSE and learning outcomes, we need to compare them to non-Waldorf students who have—at least in Austria—very limited contact to IBSE. In the present study, students of the regular PISA sample with similar sociodemographic characteristics as Waldorf students were used as the comparison group.

The present study offers new empirical evidence in two substantial respects: First, it showed that Waldorf students are more interested in science topics and report more enjoyment of learning science than other students. On the other hand, Waldorf students perform remarkably lower in science as measured by PISA than expected by their sociodemographic background. Second, we investigated if this conflicting attitude-achievement constellation of Waldorf students may be explained by a higher amount of IBSE in Waldorf education. Results revealed that Waldorf students’ more positive attitudes towards science can indeed be mainly explained by higher exposure to IBSE. However, the underperformance of Waldorf students in science may not be explained by their higher IBSE exposure. In the following we discuss these findings in more detail.

### IBSE and science achievement

Concerning science achievement, the present study showed that IBSE is not a significant mediator of the relationship between Waldorf school attendance and science achievement. Therefore Waldorf students’ underperformance in science may *not* be explained by their higher exposure to IBSE. The nearly-zero mediation indicates that a high amount of IBSE (and therefore a low amount of conventional learning methods) is about equally effective as a low amount of IBSE (and therefore a high amount of conventional learning methods) in increasing student science achievement. In terms of the effect of IBSE on achievement this shows a neutral result and does not confirm previous PISA results which showed a negative association between IBSE and achievement (OECD, 2016a) which was however very small in size. It must be noted that, in these previous results on PISA, students were not matched according to their sociodemographic background and school type. Therefore, a confounding between background characteristics and IBSE (e.g., less IBSE in high-status academic schools) may explain the more negative correlational results.

One explanation for the close-to-zero mediation could be that rather ineffective IBSE strategies might be applied in Waldorf education. For example, Jiang and McComas (2015) showed that students’ highest science achievement was obtained when they were involved in more guided conducting activities and drawing conclusions from data, rather than in more open higher level inquiry activities such as designing the investigation or raising their own questions (see also Furtak et al., 2012; Lazonder & Harmesen, 2016; Minner et al., 2010). Indeed, a very high prevalence of student investigations in science instruction has been reported by Waldorf students in PISA 2006 (Wallner-Paschon, 2009). Further studies with a closer look on the actual strategies of inquiry used in Waldorf science education may provide a clearer picture and offer further explanations on the performance of Waldorf students.

The close-to-zero mediation moreover indicates that reasons other than IBSE must contribute to the lower performance of Waldorf students. One reason may be that Waldorf students possibly differ from non-Waldorf students in other characteristics related to performance (Wallner-Paschon, 2018). For example, some Waldorf students might have changed to Waldorf schools after being denied admission to or failing out of academic-track mainstream schools (Liebenwein et al., 2012).^6^ In PISA 2015 12% of Austrian Waldorf students reported that they had formerly attended an academic-track mainstream school. As academic stratification is also related to social stratification in Austria and social background characteristics are used as covariates in PSM, matching partners of Waldorf students more often attend higher track schools (academic schools or colleges for higher vocational education) than students in the total population (76.4% and 60.9%). In these schools there is high emphasis on high achievement, first in the selective admission of students and second in the curricula. Waldorf principles however do not have a strong focus on performance (e.g., no normative achievement feedback) and students’ capabilities are often not adequately challenged (Liebenwein et al., 2012). Future studies should investigate these aspects in more detail, systematically search for possible other reasons of Waldorf students’ lower performance, and moreover control for the educational career of Waldorf students.

### IBSE and science enjoyment and interest

Concerning science enjoyment and interest, the present study showed that IBSE is a highly significant mediator of the relationship between Waldorf school attendance and science motivation. Waldorf students’ higher enjoyment of science and higher interest in science topics may almost entirely be explained by their higher exposure to IBSE. This very strong mediation effect indicates that a high amount of IBSE is much more effective than a low amount of IBSE in increasing student science attitudes. These results are in accordance with previous studies which showed a positive correlation between IBSE and science-related attitudes (Bertsch et al., 2014; Kobarg et al., 2011; Wolf & Fraser, 2008). Therefore, IBSE seems to be a highly effective strategy for boosting students’ science-related attitudes. Positive attitudes are deemed a highly important educational outcome next to achievement (Osborne, 2003; Schiepe-Tiska et al., 2016). For example, a high interest is associated with educational and professional career choices (Renninger et al., 2015). Given the importance of positive attitudes towards science, Osborne (2003, p. 1049) pointed out the urgent need “for research to identify those aspects of science teaching that make school science engaging for pupils.” The present study identified IBSE as an important aspect in this respect as it indicated that IBSE, although it may not improve achievement more than conventional educational methods, does boost enjoyment of learning science and interest in science topics. Considering the high importance of attitudes for career choice, more IBSE at school may lead to more young adults choosing science-related careers.

## Strengths, limitations, and implications

The present study is an important complement to present IBSE research. It clearly made a step beyond previous studies which rarely systematically differentiated between the achievement and attitudinal outcomes of IBSE. Moreover, existing (quasi-)experimental studies often focused on only one very specific science topic or investigated IBSE limited to the period of intervention. These students may have received little or no ISBE in their daily instruction outside the experimental situation. From Waldorf education we know that IBSE is highly integrated in day-to-day science education and not restricted to the period of experimental study. Therefore, the 15-year old Waldorf students in the present study had been taught in an IBSE-rich environment for many years (usually 10).^7^ This long period of high IBSE exposure in all aspects of science education clearly distinguishes Waldorf students from non-Waldorf students participating in (quasi-) experimental studies restricted in time and scope. Therefore the present study tells us about the long lasting and general effects of IBSE as opposed to rather short-term and specific effects of experimental studies. Of course it must be acknowledged that (quasi-) experimental studies are longitudinal in nature and test the effect of a specific IBSE intervention under controlled conditions. The present study is based on cross-sectional data and makes an attempt to imitate random assignment into a “treatment” (= Waldorf) and a “control” (= non-Waldorf) group via post-hoc matching.

A limitation of the present study is the small sample size of Waldorf students (*N* = 149). Although the present investigation is based on a census of all Austrian Waldorf students aged 15 years, there may be high variation in Waldorf student’s achievement and attitudes across different cohorts. We therefore performed generalizability analyses with the Austrian PISA 2006 sample which also included a census of 15-year-old Waldorf students (N = 153). Results showed that characteristics of Waldorf students are highly stable over cohorts. Also the differences between Waldorf and non-Waldorf students in enjoyment of science, interest in science and science achievement was quite stable from 2006 to 2015. Another measure to increase generalizability would be including a census of Waldorf students of more countries in future PISA assessments. Germany in particular would provide valuable information because of its high Waldorf population (230 schools and 85,000 students).^8^

It must be noted that PISA assessment of IBSE is very broad and does not specify the level of teacher guidance in IBSE, a crucial characteristic in rendering IBSE effective in terms of student achievement. A more sophisticated assessment of IBSE, especially with regard to IBSE-specific teacher guidance, should therefore be implemented in further PISA assessments. Moreover it is important to consider student background characteristics when investigating the relationship between IBSE and achievement (as it was done in the present study).

In addition, there are a number of variables which have not been measured in PISA but would have been valuable covariates in the PSM models, most important prior achievement but also parents’ educational goals and motives for their school choice. In this way we could control for the possibility that some students might attend a Waldorf school because their achievement was too low for an academic-track-school.

The present study was the first to demonstrate that Waldorf students show lower science achievement but more positive science attitudes than matched non-Waldorf students. Future studies should—like the present—consider the more advantageous social background of Waldorf students when comparing them to other students.

It must be noted, that the negative IBSE—science-relationship is very weak in Austria and only becomes significant after controlling for socioeconomic background (OECD, 2016a, p. 303). Therefore, it is unclear if the result that IBSE has no detrimental effects on achievement can be generalized to other countries like New Zealand, Greece or Estonia where the negative relationship is remarkably stronger than in Austria.

## Conclusions

Attending a school type with a high level of IBSE may have positive effects on attitudinal outcomes (enjoyment in learning science and interest in broad science topics) whereas it does not seem to have notable effects on science achievement. This indicates that IBSE could be applied in educational contexts aiming to increase students’ scientific attitudes. Scientific attitudes have already been shown to be crucial if the goal is to attract more young people to science and science-related jobs. Therefore IBSE could help us not only to increase the number of students who are excited about science but also of adults who follow a science career.

## Data Availability

PISA 2018 data (including all Austrian data) is available under https://www.oecd.org/pisa/data/2018database/. IDs of Waldorf schools are available from the IQS upon request (fdb@iqs.gv.at).

## Abbreviations

IBSE: Inquiry-based science education
ISCED: International Standard Classification of Education
MLR: Maximum likelihood with robust standard errors
NSES: United States National Science Education Standards
OECD: Organisation for Economic Co-operation and Development
PVs: Plausible values
PISA: Programme for International Student Assessment
SEM: Structural equation modeling
SES: Socioeconomic status
WLEs: Weighted likelihood estimates

## Footnotes

1 Only Austrian schools are included here because our study only includes Austrian data (see “Methods” section).

2 The target population of PISA 2015 in Austria was students born between August 1, 1999 und July 31, 2000. Therefore, 68% of the tested students were 15 years old, and 32% were 16 years old at the time of the test. For simplicity reasons, we refer to 15-year olds throughout this manuscript.

3 Downloaded from https://www-01.ibm.com/marketing/iwm/iwm/web/reg/pick.do?source=swg-tspssp&lang=en_US.

4 Downloaded from https://sourceforge.net/projects/psmspss/.

5 Nearest neighbor matching selects the closest eligible non-Waldorf student to be paired with each Waldorf student.

6 Austria has a highly stratified education system. Between-school tracking starts after fourth grade, and admission to higher-track academic schools is dependent upon previous achievement.

7 Most Austrian students tested in PISA 2015 are at the end of Grade 10 (71%). It must be noted that some Waldorf students are lateral entrants and have not passed their entire school career in Waldorf schools (Liebenwein et al., 2012). In PISA 2015 19% of Austrian Waldorf students reported that they had not only attended a Waldorf school since primary school.

8 https://www.welt.de/print/die_welt/wissen/article109490665/Wissenschaftler-loben-Waldorfschulen.html.
